# Changes in foot volume, body composition, and hydration status in male and female 24-hour ultra-mountain bikers

**DOI:** 10.1186/1550-2783-11-12

**Published:** 2014-03-24

**Authors:** Daniela Chlíbková, Beat Knechtle, Thomas Rosemann, Alena Žákovská, Ivana Tomášková, Marcus Shortall, Iva Tomášková

**Affiliations:** 1 Centre of Sports Activities, Brno University of Technology, Brno, Czech Republic; 2 Institute of General Practise and for Health Services Research, University of Zurich, Zurich, Switzerland; 3 Institute of Experimental Biology, Faculty of Science, Masaryk University, Brno, Czech Republic; 4 Faculty of Forestry and Wood Sciences, Czech University of Life Sciences, Prague, Czech Republic; 5 Institute of Technology Tallaght, Dublin, Ireland; 6 SurGal clinic s.r.o., Center for Sports Medicine, Brno, Czech Republic

**Keywords:** Body mass, Fat mass, Hydration, Foot volume

## Abstract

**Background:**

The effects of running and cycling on changes in hydration status and body composition during a 24-hour race have been described previously, but data for 24-hour ultra-mountain bikers are missing. The present study investigated changes in foot volume, body composition, and hydration status in male and female 24-hour ultra-mountain bikers.

**Methods:**

We compared in 49 (37 men and 12 women) 24-hour ultra-mountain bikers (ultra-MTBers) changes (Δ) in body mass (BM). Fat mass (FM), percent body fat (%BF) and skeletal muscle mass (SM) were estimated using anthropometric methods. Changes in total body water (TBW), extracellular fluid (ECF) and intracellular fluid (ICF) were determined using bioelectrical impedance and changes in foot volume using plethysmography. Haematocrit, plasma [Na^+^], plasma urea, plasma osmolality, urine urea, urine specific gravity and urine osmolality were measured in a subgroup of 25 ultra-MTBers (16 men and 9 women).

**Results:**

In male 24-hour ultra-MTBers, BM (*P* < 0.001), FM (*P* < 0.001), %BF (*P* < 0.001) and ECF (*P* < 0.05) decreased whereas SM and TBW did not change (*P* > 0.05). A significant correlation was found between post-race BM and post-race FM (r = 0.63, *P* < 0.001). In female ultra-MTBers, BM (*P* < 0.05), %BF (*P* < 0.05) and FM (*P* < 0.001) decreased, whereas SM, ECF and TBW remained stable (*P* > 0.05). Absolute ranking in the race was related to Δ%BM (*P* < 0.001) and Δ%FM in men (*P* < 0.001) and to Δ%BM (*P* < 0.05) in women. In male ultra-MTBers, increased post-race plasma urea (*P* < 0.001) was negatively related to absolute ranking in the race, Δ%BM, post-race FM and Δ%ECF (*P* < 0.05). Foot volume remained stable in both sexes (*P* > 0.05).

**Conclusions:**

Male and female 24-hour ultra-MTBers experienced a significant loss in BM and FM, whereas SM remained stable. Body weight changes and increases in plasma urea do not reflect a change in body hydration status. No oedema of the lower limbs occurred.

## Background

Ultra-endurance races defined as an event exceeding six hours in duration and lasting up to 40 hours or several days [1] pose specific problems for competitors such as a possibility of lack of fluids [2-6], fluid overload and/or an increase in total body water [4,7-17], sleep deprivation [2,18-21], inadequate energy intake [2,15,21-24] or unfavorable conditions like extreme heat or extreme cold [2,5,7,12,16,25,26]. Issues associated with body composition and hydration status include a decrease in body mass in ultra-running [2,9,16,27-29], in road ultra-cycling [21,22,24], in mountain-biking [5,7,30], swimming [12,31], triathlon [6,15,32] and skiing [26].

Within ultra-races, there is a difference between single stage races [30,33-37], multi-stage races [7,22,25,33,38-40] and time-limited races such as 24-hour races [2,16,18-21,27-29,41]. Little is known about the effects of running or cycling on changes in hydration status [16,28,41] and body composition [2,16,18,20,27,29] during a 24-hour race. Non-stop ultra-endurance races and races lasting for several days without defined breaks lead generally to a decrease in body mass [15,22,24], and there seemed to be differences between cycling and running races. A decrease in fat mass has been rather reported for ultra-cycling [5,21,22,24,36], whereas a decrease in skeletal mass has been more often reported for ultra-running [17,42]. However, a reduction in fat mass has not been confirmed for a 24-hour cycling road race. Knechtle et al. [20] showed that an energy deficit did not always result in a reciprocal loss of adipose subcutaneous tissue or skeletal muscle mass.

A decrease in body mass could also be attributed to dehydration [2,5], but dehydration cannot be established without the determination of plasma sodium concentration [Na^+^] or osmolality in both plasma and urine [43]. Male ultra-MTBers during a 120-km race suffered a significant decrease in both body mass and skeletal mass, but no dehydration was observed when other determinants of hydration status were assessed [30]. On the contrary, body mass can increase [13,23] or remain stable [25,42] in ultra-endurance races with breaks due to an increase in total body water.

An increase in total body water can occur in several ways such as fluid overload [8,9], plasma [Na^+^] retention [30] due to an increased aldosterone activity [34], protein catabolism [6], an increased vasopressin activity [44] or an impaired renal function [17,45]. Prolonged strenuous endurance exercise may lead to an increase in extracellular fluid, plasma volume and total body water [8,10,17] and a decrease in haematocrit due to haemodilution [7]. For male 100-km ultra-runners, a loss of both skeletal muscle mass and fat mass with an increase in total body water has been reported [46]. Similar findings were recorded in a Triple Iron ultra-triathlon (*i.e.* 11.4 km swimming, 540 km cycling, and 126.6 km running) where total body water and plasma volume increased and these changes seemed to be associated with oedema of the feet [10]. Two field studies using plethysmography found a potential association between fluid intake and the formation of peripheral oedema [8,9].

Moreover, only a few studies investigated changes in body composition and hydration status in female ultra-endurance athletes [12,41,47-52], but the reported findings were not consistent. In open-water ultra-distance swimmers, Weitkunat et al. [12] summarized that changes in body composition and hydration status were different in male compared to female athletes. For ultra-marathoners, it has been shown that female runners lost body mass during a 24-hour run [41]. Knechtle et al. [47] observed in 11 female 100-km ultra-runners a loss in body mass despite unchanged total body water and plasma [Na^+^]. On the contrary, in one female ultra-runner during a 1,200-km multi-stage ultra-marathon, body mass increased, percent body fat decreased, while percent total body water and skeletal mass increased [51].

Additionally, there are no studies showing whether changes in body composition and hydration status were associated with an increased prevalence of peripheral oedema in ultra-endurance mountain bikers such as 24-hour ultra-MTBers. The aim of the present study was therefore to investigate changes in foot volume, body composition and hydration status in male and female 24-hour ultra-MTBers. Based on present literature, we hypothesized to find a loss in body mass as has previously reported for ultra-cycling [21,24,36] and non-stop ultra-endurance races [15,22,24,26]. We hypothesized that this type of MTB races would lead to an increase in foot volume due to peripheral oedema.

## Methods

### Participants

The present work combines data from two 24-hour races held in the Czech Republic in 2012. Subjects were recruited via pre-race emails and during race registration. A total of 28 (22 men and 6 women) recreational 24-hour ultra-MTBers in the solo category from the ‘Czech Championship 24-hour MTB 2012’ in Jihlava city in the Czech Republic and 24 (18 men and 6 women) ultra-MTBers from the ‛Bike Race Marathon MTB Rohozec 24 hours’ in Liberec city in the Czech Republic in the solo category consented to participate in the study. Of those, 37 men and 12 women finished the race successfully. One cyclist had to give up due to technical problems and two athletes because of medical complications. Athletes were informed that participation was voluntary and that the project had received approval in accordance with the law (No. 96/2001 Coll. M. S. on Human Rights and Biomedicine and Act No. 101/2000 Coll. Privacy). The pre-race anthropometry and training data of the participants are presented in Table 1.

**Table 1:** The pre-race experience and training parameters (n = 49)

	**Male ultra-MTBers**	**Female ultra-MTBers**
	**(n = 37)**	**(n = 12)**
	***M*** **±** ***SD***	***M*** **±** ***SD***
Years as active biker (yr)	9.2 ± 5.8	8.8 ± 5.9
Number of finished ultra-marathons (n)	8.0 ± 6.5	6.7 ± 5.3
Personal best km in 24 hour (km)	315.5 ± 89.7	279.6 ± 106.7
Total hours weekly (h)	10.5 ± 5.3	10.2 ± 5.5
Weekly cycling kilometers (km)	225.8 ± 149.5	191.8 ± 134.5
Weekly cycling hours (h)	9.9 ± 5.1	9.2 ± 5.2
Mean cycling intensity (beat/min)	133.8** ± 7.6	134.5** ± 22.8
Mean cycling speed (km/h)	23.0** ± 3.6	21.1** ± 5.3
Longest trail (km)	176.8** ± 84.7	141.7** ± 75.5
Amount of km in 2011 (km)	7,107.5 ± 5,782.4	5,696.9 ± 5,037.9

Results are presented as mean ± SD; * = *P* < 0.05, ** = *P* < 0.001.

### Races details

The first measurement was performed at the 3rd edition of the ‘Czech Championship 24-hour MTB 2012’ in Jihlava. The ultra-MTBers began the race at 12:00 on 19th May 2012 and finished at 12:00 on 20th May 2012. The course comprised a 9.5 km single-track with an elevation of 220 m. A single aid station, located at the start/finish area was provided by the organizer where a variety of food and beverages such as hypotonic sports drinks, tea, soup, caffeinated drinks, water, fruit, vegetables, energy bars, bread, soup, sausages, cheese, bread, chocolate and biscuits were available. The ultra-MTBers could also use their own supplies in their pit stops. Temperature was +16˚C at the start, rose to a maximum of +20˚C, dropped to +6˚C during the night and rose to +23˚C from the morning of the next day till the end of the race. Cloud cover was minimal and no precipitation was recorded during the race. The relative humidity was stable at 43% during the race. The ‘Bike Race Marathon MTB Rohozec’ in Liberec took place from 9th June to 10th June 2012. The course comprised a 12.6 km track with an elevation of 250 m. The track surface consisted of paved and unpaved roads and paths. There was one aid station located at the start and finish area with food and beverages similar to those mentioned above. The temperature was +19˚C at the start, rose to a maximum of +23˚C, dropped to +6˚C during the night and changed to +11˚C until the end of the race. Weather conditions varied from sunny to cloudy with a short shower in the afternoon and relative humidity increased from 44% to 98%.

### Procedures, measurements and calculations

Participants were instructed to keep a training diary until the start of the race. The training three months before the race (*i.e.* training units in hours, cycling units in hours, training distances in kilometers, cycling speed, heart rate during training units, volume of kilometers in the year 2011, and the years of active cycling) was recorded. Participant recruitment and pre-race testing took place during event registration in the morning before the race between 07:00 a.m. and 11:00 a.m. in a private room adjacent to the registration area. The athletes were informed of the procedures and gave their informed written consent. Post-race measurements were taken between 12:00 and 1:00 p.m. immediately upon completion of the race in the same place. No measurements were made during the race. Between the pre- and the post-race measurements, all athletes recorded their fluid intake using a written record.

### Anthropometric measurements and plethysmography of the foot

**Figure 1:**
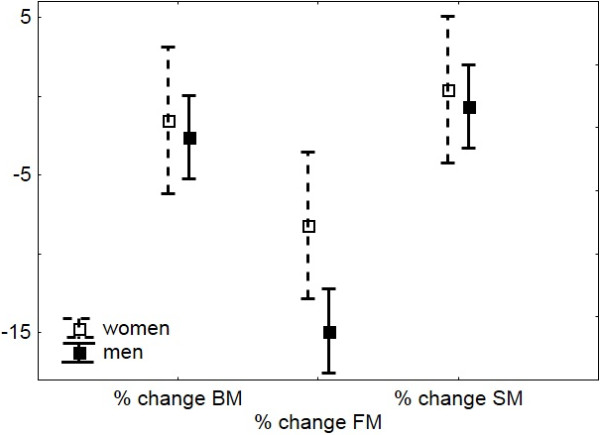
**Absolute ranking related to %ΔBM and fluid intake in men (n = 37) and women (n = 12).** Absolute ranking – according to the number of achieved kilometers during 24 hours, %ΔBM – percent change in body mass.

Anthropometric measurements were recorded in all forty-nine ultra-MTBers (37 males and 12 females) (Table 2, also Figure 1) to estimate skeletal muscle mass and fat mass. Body mass, total body water, extracellular fluid and intracellular fluid were measured using a multiple-frequency bioelectrical impedance analyser (InBody 720, Biospace, Seoul, South Korea). Inbody 720 has a tetra polar 8-point tactile electrode system performing at each session 30 impedance measurements by using six different frequencies (*i.e.* 1 kHz, 5 kHz, 50 kHz, 250 kHz, 500 kHz, and 1,000 kHz) at each five segments (*i.e.* right arm, left arm, trunk, right leg, and left leg). Subjects were barefoot and generally clothed in cycling attire for both the pre- and post-race measurements and participants were advised to void their urinary bladder prior to the anthropometric measurements. Body height was determined using a stadiometer (TANITA HR 001, Tanita Europe B.V., Amsterdam, The Netherland) to the nearest 0.01 m. Body mass index was calculated using body mass and body height. The circumferences of mid-upper arm, mid-thigh and mid-calf were measured on the right side of the body to the nearest 0.01 cm using a non-elastic tape measure (KaWe CE, Kirchner und Welhelm, Germany). The skin-fold measurements were taken on the right side of the body for all eight skin-folds (*i.e.* pectoralis, axillar, triceps, subscapular, abdomen, suprailiac, front thigh, and medial calf) using a skin-fold calliper (Harpenden skinfold caliper, Baty International Ltd) and recorded to the nearest 0.2 mm. An anthropometric equation [53] using body stature, corrected upper arm and thigh girth, sex, age and race of the participants was used to estimate skeletal muscle mass in kg. Fat-free mass (kg) was estimated using an equation for male [54] and female [55] athletes. Fat mass (kg) was determined based on subtracting fat-free mass from total body mass. Percent body fat was estimated using a specific equation for men [56] and women [57]. Hydration status was classified according to the criteria established by Noakes et al. [11] with overhydration classified as any weight gain above initial body mass, euhydration as a decrease in body mass of 0.01% to 3.0%, and dehydration as any decrease in body mass greater than 3.0%. The changes of the volume of the right foot were estimated using the principle of plethysmography [8]. We used a Plexiglas vessel, the dimensions were chosen so that any foot size of an ultra-MTBer would fit in the vessel. Outside the vessel, a scale in mm was fixed on the front window to measure changes in the level of water from the bottom to the top. The vessel was filled to the level of 100 mm with tap water. The right foot was immersed in the water and the upper limit of the water was at the middle of *malleolus medialis*. After immersion of the foot, the new water level was recorded to the nearest 1 mm and the volume of the foot was calculated. The corresponding calculated volume in ml using the length, width and height in mm of the displaced water was defined as the volume of the right foot. No measurements were made during the race.

**Table 2:** Age and anthropometric characteristics of the ultra-MTBers (n = 49)

**Parameter**	**Pre-race**	**Post- race**	**Absolute change**	**Change (%)**
	***M*** **±** ***SD***	***M*** **±** ***SD***		
**Male ultra-MTBers (n = 37)**				
Body height (cm)	180.4 ± 0.1			
Age (yr)	36.6 ± 8.4			
Body mass (kg)	77.9 ± 9.6	75.9 ± 9.8	-2.0 ± 1.6**	-2.6 ± 2.1**
Skeletal muscle mass (kg)	38.4 ± 4.9	38.1 ± 4.9	-0.3 ± 1.1	-0.6 ± 2.7
Fat mass (kg)	10.6 ± 5.3	9.2 ± 4.9	-1.4 ± 1.2**	-14.9 ± 14.5**
Percent body fat (%)	13.2 ± 5.7	11.8 ± 5.4	-1.4 ± 1.4**	-12.7 ± 14.6**
Total body water (L)	49.3 ± 5.5	48.9 ± 5.7	-0.4 ± 1.4	-0.9 ± 2.8
Extracellular fluid (L)	18.3 ± 2.0	18.1 ± 2.1	-0.2 ± 0.6*	-1.2 ± 3.2*
Intracellular fluid (L)	31.0 ± 3.5	30.8 ± 3.6	-0.2 ± 0.8	-0.7 ± 2.6
Volume of the foot (L)	1.132 ± 1.502	1.145 ± 1.302	0.013 ± 0.097	1.8 ± 9.6
**Female ultra-MTBers (n = 12)**				
Body height (cm)	167.8 ± 29.3			
Age (yr)	36.8 ± 8.9			
Body mass (kg)	60.6 ± 4.9	59.7 ± 4.9	-0.9 ± 1.2*	-1.5 ± 1.9*
Skeletal muscle mass (kg)	26.7 ± 3.3	26.8 ± 3.2	0.1 ± 0.7	0.4 ± 2.7
Fat mass (kg)	10.9 ± 3.9	9.7 ± 3.9	-1.2 ± 1.0**	-8.2 ± 10.8**
Percent body fat (%)	15.4 ± 6.5	13.7 ± 6.2	-2.7 ± 3.6*	-11.0 ± 15.5*
Total body water (L)	35.3 ± 4.4	35.4 ± 4.5	0.1 ± 0.9	0.2 ± 2.7
Extracellular fluid (L)	13.3 ± 1.7	13.3 ± 1.7	0.0 ± 0.5	0.0 ± 3.6
Intracellular fluid (L)	22.0 ± 2.7	22.1 ± 2.8	0.1 ± 0.5	0.4 ± 2.3
Volume of the foot (L)	0.858 ± 1.205	0.908 ± 1.100	0.050 ± 0.116	6.9 ± 14.4

Results are presented as mean ± SD; * = *P* < 0.05, ** = *P* < 0.001.

### Haematological and biochemical measurements

Haematocrit (HCT), plasma sodium [Na^+^], plasma urea, plasma osmolality, urine urea, urine specific gravity (USG) and urine osmolality pre- and post-race measurements were determined in a subgroup of twenty-five athletes (16 men and 9 women) to investigate changes in hydration status (Table 3). These procedures were performed at the same time as the anthropometric measurements, before the start and directly after finishing the race. The recording procedure for pre- and post-race measurements was identical. After venipuncture of an antecubital vein, one Sarstedt S-Monovette (plasma gel, 7.5 mL) for chemical and one Sarstedt S-Monovette (EDTA, 2.7 mL) for haematological analysis were cooled and sent to the laboratory and were analysed within six hours. Haematocrit was determined using Sysmex XE 2100 (Sysmex Corporation, Japan), plasma [Na^+^] and plasma urea using a biochemical analyzer Modula SWA, Modul P + ISE (Hitachi High Technologies Corporation, Japan, Roche Diagnostic), and plasma osmolality using Arkray Osmotation (Arkray Factory, Inc., Japan). Samples of urine were collected in one Sarstedt monovette for urine (10 mL) and sent to the laboratory. Urine urea was determined using a biochemical analyzer Modula SWA, Modul P + ISE (Hitachi High Technologies Corporation, Japan, Roche Diagnostic), urine specific gravity using Au Max-4030 (Arkray Factory, Inc., Japan), and urine osmolality using Arkray Osmotation (Arkray Factory, Inc., Japan).

**Table 3:** Haematological and urinary parameters (n = 25)

**Parameter**	**Pre-race**	**Post-race**	**Absolute change**	**Change (%)**
	**M ± SD**	**M ± SD**		
**Male ultra-MTBers(n = 16)**				
Haematocrit (%)	43.1 ± 3.3	42.6 ± 3.1	-0.5 ± 3.7	-0.7 ± 8.8
Plasma sodium (mmol/L)	138.2 ± 1.4	137.8 ± 2.3	-0.4 ± 2.9**	-0.3 ± 2.1
Plasma urea (mmol/L)	6.1 ± 1.3	13.5 ± 4.1	7.4 ± 3.8**	124.0 ± 67.2
Plasma osmolality (mosmol/kg H_2_O)	289.4 ± 4.1	293.6 ± 4.4	4.2 ± 4.5**	1.5 ± 1.6
Urine urea (mmol/L)	239.3 ± 172.1	576.0 ± 78.0	336.7 ± 174.8**	298.0 ± 315.5
Urine osmolality (mosmol/kg H_2_O)	415.7 ± 190.3	776.7 ± 133.4	361.0 ± 184.4**	132.0 ± 132.4
Urine specific gravity (g/mL)	1.013 ± 0.002	1.022 ± 0.004	0.009 ± 0.004**	0.8 ± 0.3
**Female ultra-MTBers (n = 9)**				
Haematocrit (%)	42.0 ± 2.7	40.0 ± 2.8	-2.0 ± 4.1	-4.5 ± 10.0
Plasma sodium (mmol/L)	137.4 ± 2.8	137.1 ± 1.8	-0.3 ± 3.0	-0.2 ± 2.2
Plasma urea (mmol/L)	5.8 ± 1.5	8.7 ± 2.5	2.9 ± 1.2**	46.9 ± 18.5
Plasma osmolality (mosmol/kg H_2_O)	292.2 ± 2.8	290.6 ± 4.6	-1.7 ± 4.3	-0.6 ± 1.5
Urine urea (mmol/L)	290.5 ± 204.9	463.0 ± 172.5	172.5 ± 246.5	190.6 ± 292.3
Urine osmolality (mosmol/kg H_2_O)	724.3 ± 214.0	716,4 ± 329.1	-7.9 ± 276.5	-1.0 ± 36.6
Urine specific gravity (g/mL)	1.000 ± 0.005	1.001 ± 0.005	0.001 ± 0.005	0.1 ± 0.4

Results are presented as mean ± SD; * = *P* < 0.05, ** = *P* < 0.001.

### Statistical analysis

Results are presented as mean ± standard deviation (SD). The Shapiro-Wilk test was applied to check for normal distribution of data. Differences between men and women in parameters of pre-race experience and training, the average race speed and the total number of kilometers were evaluated using paired *t*-test. The correlations of the changes in parameters during the race were evaluated using Pearson product–moment in male group and Spearman correlation analysis to assess uni-variate associations in female group. Paired *t*-tests in male group and the Wilcoxon signed rank tests in female group were used to check for significant changes in the anthropometric and laboratory parameters before and after the race. The critical value for rejecting the null hypothesis was set at 0.05. The data was evaluated in the program Statistic 7.0 (StatSoft, Tulsa, U.S.A.).

## Results

### Pre-race experience and training parameters

Pre-race results of 37 male and 12 female 24-hour ultra-MTBers are presented in Table 1. Male ultra-MTBers displayed a significantly higher body stature and body mass compared to female ultra-MTBers. Additionally, mean training cycling intensity, mean training cycling speed and session duration during pre-race training were higher in men compared to women. On the contrary, no significant differences between sexes were noted in the years spent as an active MTBer, in the number of finished ultra-cycling marathons, in the personal best performance in a 24-hour cycling race, in total hours spent cycling in training, in the total duration (hour) and the distance (km) of a cycling training in the three months before the race.

### Race performance and changes in body composition

Forty-nine ultra-MTBers (37 men and 12 women) finished the race. Significant differences in the average cycling speed during the race were observed between male (16.7 ± 2.2 km/h) and female (14.2 ± 1.7 km/h) ultra-MTBers (*P* < 0.001). Men achieved a mean distance of 282.9 ± 82.9 km during the 24 hours, whereas women achieved 242.4 ± 69.6 km. Despite the differences in the average speed for each sex, men did not achieve a significantly higher number of kilometers during the 24 hours (*P* > 0.05).

In men, the change in body mass was significantly and negatively related to the achieved number of kilometers during the 24 hours (r = -0.41, *P* < 0.05). Their absolute ranking in the race was significantly and positively related to post-race body mass (r = 0.40, *P* < 0.05), the change in body mass (r = 0.46, *P* < 0.001), the percent change in body mass (r = 0.50, *P* = 0.001) (Figure 1) and the percent change in fat mass (r = 0.44, *P* < 0.001) and significantly and negatively related to fluid intake (r = -0.54, *P* < 0.05) (Figure 1) and percent change in plasma urea (r = -0.53, *P* < 0.05). Men’s’ absolute ranking in the race was not related to changes in plasma [Na^+^], or percent changes in urine specific gravity (*P* > 0.05).

Changes in body mass were significantly and negatively related to the number of achieved kilometers during the 24 hours also in women (r = -0.80, *P* < 0.001). Their absolute ranking during the race was significantly and positively related to the change in body mass (r = 0.70, *P* < 0.05), the percent change in body mass (r = 0.77, *P* < 0.05) (Figure 1), and significantly and negatively related to fluid intake (r = -0.73, *P* < 0.05) (Figure 1) during the race. Women’ absolute ranking in the race was not related to percent change in fat mass, or percent change in urine specific gravity (*P* > 0.05).

### Changes in body composition with regard to anthropometric, urine and blood measurements

The correlation matrix of post-race body mass, change in body mass, percent change in body mass, post-race fat mass, percent change in fat mass, percent change in extracellular fluid and percent change in plasma urea for men is shown in Table 4. The correlation matrix of change in body mass, percent change in body mass and percent change in fat mass for women is presented in Table 5.

**Table 4:** Correlation matrix of PR BM, ΔBM, %ΔBM, PR FM, %ΔFM, %ΔECF and %Δ plasma urea for men (n = 37)

PR BM	0.20	0.33*	0.63**	0.17	0.35*	-0.10
	ΔBM	0.99**	0.19	0.30	0.88**	-0.44
		%ΔBM	0.53*	0.33*	0.83**	-0.50*
			PR FM	0.45**	0.29	-0.53*
				%ΔFM	-0.05	-0.31
					%ΔEXW	-0.52*
						%ΔPU

PR BM – post-race body mass, ΔBM – change in body mass, %ΔBM – percent change in body mass, PR FM – post-race body mass, %ΔFM – percent change in fat mass, %ΔECF – percent change in extracellular fluid, %Δ plasma urea – percent change in plasma urea. Output file contain both the Pearson’s r values and the scatter plot, one star (*) above the Pearson value represents significance level *P* < 0.05, two stars (**) *P* < 0.001.

**Table 5:** The correlation matrix of ΔBM, %ΔBM and %ΔFM for women (n = 12)

ΔBM	0.99**	0.35
	%ΔBM	0.36
		%ΔFM

ΔBM – change in body mass, %ΔBM – percent change in body mass, %ΔFM – percent change in fat mass. Output file contain both the Spearman’s rank correlation coefficient and the scatter plot, one star (*) above the Spearman value represents significance level *P* < 0.05, two stars (**) *P* < 0.01.

**Figure 2:**
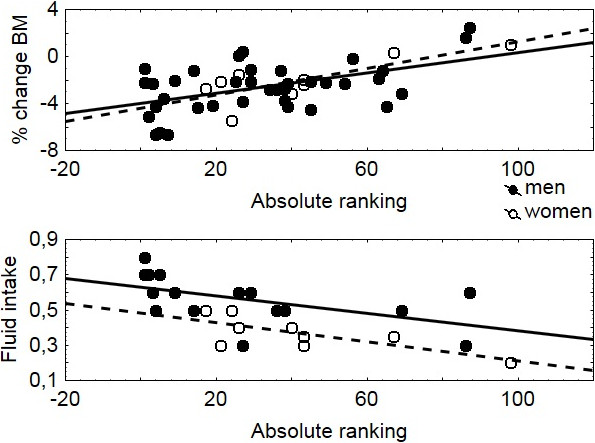
**Percentage change of BM, FM, and SM in the 37 men and 12 women during the 24 hour MTB race.** BM – body mass, FM – fat mass, SM – skeletal muscle mass.

In male ultra-MTBers (n = 37) body mass decreased significantly during the race by 2.0 ± 1.6 kg, equal to 2.6 ± 2.1% (*P* < 0.001) (Table 2, also Figure 2). Fat mass decreased significantly by 1.4 ± 1.2 kg (*P* < 0.001), percent body fat decreased significantly by 1.4 ± 1.4% (*P* < 0.001), whereas skeletal muscle mass decreased non-significantly by 0.6 ± 2.7% (*P* > 0.05) (Table 2, also Figure 2). In men, post-race body mass was significantly and positively related to post-race fat mass (r = 0.63, *P* < 0.001). Percent changes in body mass were significantly and positively related to post-race fat mass (r = 0.53, *P* < 0.05) and percent changes in skeletal muscle mass (r = 0.73, *P* < 0.001) (Table 4). The change in body mass was neither related to the change in plasma [Na^+^], nor to the percent change in urine specific gravity (*P* > 0.05).

For men, the percent changes in haematocrit remained stable, and plasma volume increased non-significantly by 3.5% (14.8%). Plasma [Na^+^] in male ultra-MTBers decreased significantly (*P* < 0.001) by 0.3% from 138.2 mmol/L pre-race to 137.8 mmol/L post-race (Table 3). Urine specific gravity increased significantly (*P* < 0.001) (Table 3). Changes in plasma [Na^+^] were not related to percent changes in urine specific gravity (*P* > 0.05). Post-race plasma osmolality increased significantly (*P* < 0.001) (Table 3), but was not related to the changes in body mass, plasma [Na^+^], urine osmolality, or urine urea (*P* > 0.05). Percent changes in urine osmolality were not related to percent changes in urine urea. Percent changes in plasma urea were significantly and positively related to post-race plasma osmolality (r = 0.49, *P* < 0.05), and significantly and negatively to percent changes in body mass (r = -0.50, *P* < 0.05), post-race fat mass (r = -0.53, *P* < 0.05) and percent changes in skeletal mass (r = -0.51, *P* < 0.05) (Table 4). Post-race plasma urea or the changes in plasma urea were not related to percent changes in urine specific gravity (*P* > 0.05).

In females ultra-MTBers (n = 12), body mass decreased by 0.9 ± 1.2 kg, equal to 1.5 ± 1.9% (*P* < 0.05) (Table 2, also Figure 2). Fat mass decreased significantly by 1.2 ± 1.2 kg (*P* < 0.001), percent body fat decreased by 2.7 ± 3.6% (*P* < 0.05) whereas skeletal muscle mass remained stable (*P* > 0.05) (Table 2, also Figure 2). The percent changes in body mass were not related to post-race fat mass (*P* > 0.05), or fluid intake (*P* > 0.05). Percent changes in body mass were significantly and positively related to percent changes in skeletal muscle mass (r = -0.59, *P* < 0.05), however, skeletal muscle mass did not change significantly (*P* > 0.05). The changes in body mass were not related to percent changes in urine specific gravity. The percent change in haematocrit remained stable post-race (*P* > 0.05). Plasma volume increased non-significantly by 5.6% (13.5%) (*P* > 0.05) and was not associated with percent changes in total body water, extracellular fluid or intracellular fluid (*P* > 0.05). Plasma urea increased significantly (*P* < 0.001) (Table 3). The changes in plasma urea were not related to the changes in body mass, fat mass, or in urine specific gravity (*P* > 0.05). Post-race plasma [Na^+^], plasma and urine osmolality and urine urea remained stable (*P* > 0.05).

### Changes in body water, fluid intake, and foot volumes

The correlation matrix of post-race body mass, changes in body mass, percent change in body mass, post-race fat mass, percent change in fat mass, percent change in extracellular fluid and percent change in plasma urea is shown for men in Table 4.

The male group (n = 37) consumed a total of 13.4 L of fluids during the race, equal to 0.6 ± 0.1 L/h. Fluid intake varied between 0.30 L/h and 0.80 L /h. Fluid intake was not related to changes in body mass, fat mass, extracellular fluid, plasma urea or post-race plasma [Na^+^] (*P* > 0.05). Extracellular fluid decreased by 0.2 ± 0.6 L (*P* < 0.05), whereas total body water and intracellular fluid decreased non-significantly in men (*P* > 0.05) (Table 2). Percent changes in extracellular fluid were significantly and positively related to changes in body mass (r = 0.88, *P* < 0.001), and significantly and negatively to percent changes in plasma urea (r = -0.52, *P* < 0.05). On the contrary, percent changes in extracellular fluid were not associated with percent changes in plasma volume or fluid intake. The volume of the lower leg remained unchanged in men (*P* > 0.05) (Table 2), and was neither related to fluid intake nor to changes in plasma [Na^+^] (*P* > 0.05). The male 24-hour ultra-MTBers were on average euhydrated post-race (Table 2). Thereof, twenty male ultra-MTBers were euhydrated (54.2%), thirteen were dehydrated (35.1%), and four males were overhydrated (10.7%) following the definition of Noakes et al. [11].

The female group (n = 12) consumed a total of 8.88 L of fluids during the race, equal to 0.37 L/h. Fluid intake varied between 0.20 L/h and 0.50 L/h. Fluid intake was not related to percent changes in body mass, changes in fat mass, or changes in plasma urea (*P* > 0.05). The volume of the lower leg remained unchanged in women (*P* > 0.05) (Table 2), and was neither related to fluid intake nor to changes in plasma [Na^+^] (*P* > 0.05). The female ultra-MTBers were on average euhydrated (Table 2). Thereof, seven female ultra-MTBers were euhydrated (58.3%), two were dehydrated (16.7%) and three were overhydrated (25.0%) following the definition of Noakes et al. [11].

## Discussion

The first important finding of this study was that both male and female 24-hour ultra-MTBers suffered significant losses in body mass and fat mass during the 24-hour MTB race. Skeletal muscle mass showed, however, no significant changes in contrast to fat mass. The second important finding for men was that changes in body mass were related to a decrease in post-race fat mass, and correlated with the changes in extracellular fluid and post-race plasma urea. The third important finding was that the volume of the lower leg remained unchanged in both men and women and was neither related to fluid intake nor to the changes in plasma [Na^+^]. And a last finding was that faster men and women drank more than the slower ones and showed higher losses in body mass, in men also higher fat mass losses. However, fluid intake was not correlated to changes in body mass.

### Decrease in total body mass

Changes in body mass reached statistical significance (*P* < 0.05) for both male and female 24-hour ultra-MTBers. Compared to women, men’s average decrease in body mass was 1.1 percent points (pp) lower. In ultra-endurance settings where athletes race for hours, days, or weeks without a break during the night, a decrease of body mass is a common finding, in which both fat mass and skeletal muscle mass seemed to decrease [2,6,22,24,26].

Changes in fat mass in male and female ultra-MTBers were heterogeneous and did not reach statistical significance (*P* > 0.05). Nevertheless, men’s change in fat mass was 6.7 pp lower and was related to a decrease in body mass. A better explanation of the higher changes of body mass and fat mass in men could be the reason that their pre-race values of body mass were higher than in women, men were faster than women and also the substrate utilisation during submaximal exercise in endurance-trained athletes differs between the sexes [23,58], where the contribution of intramyocellular lipids to energy supply during endurance performance could be higher in men compared to women. A decrease in fat mass is expected in an ultra-endurance performance of approximately two days [26]. Studies on ultra-triathletes [59] and ultra-cyclists [36] reported a decrease in fat mass. The 24-hour ultra-MTBers in the present study had to continuously perform for nearly 24 hours, which might explain their great losses in both body mass and fat mass. We assume that adipose subcutaneous tissue was the main energy source for a long-lasting performance such as a 24-hour MTB race and the ability to use body fat as fuel is important in a such a type of ultra-endurance performance [23,26].

In the present study, skeletal muscle mass showed no statistically significant changes in both male and female ultra-bikers. Skeletal muscle mass decreased in ultra-endurance races without breaks [22,24]. An excessive increase in endurance activities might lead to a reduction in skeletal muscle mass [12,31]. However, a loss in skeletal muscle mass might be dependent upon race intensity and was not reported for all endurance sports [12]. The decrease in skeletal muscle mass has been demonstrated rather in case reports [15,22,24] than in field studies [27,44,60], and a decrease in body mass was mainly due to a decrease in fat mass [22,24,26] than in skeletal muscle mass, such as in the present study.

Furthermore, in a study of an ultra-cycling race over 230 km with 5,500 m of altitude no evidence of exercise-induced skeletal muscle damage was reported [37]. In another study of a 600-km cycling race, again no decrease in skeletal muscle mass was found [36]. Cycling involves predominantly concentric muscle activity which will not lead to skeletal muscle damage, which may explain the lack of skeletal muscle mass loss in cyclists [39,61]. In general, we assume that a 24-hour MTB race may rather lead to a reduction of adipose subcutaneous tissue as has been reported in other studies [23,26], due to the fact that fatty acids of adipose subcutaneous tissue are oxidized in the contracting skeletal muscle [62]. Also low temperatures during night could increase carbohydrate metabolism, especially when shivering [63]. The reduction of glycogen stores along with glycogen-bound water [46,59] would result also in a loss of body mass. It is likely that the present male and female 24-hour ultra-MTBers started the race with full glycogen stores in both skeletal muscles and liver and the stores decreased during the race. We presume that the decrease in body mass could be the result of the metabolic breakdown of fuel, which includes a loss of fat, glycogen and water stored with glycogen. It is possible that the 24-hour race format may lead to a large energy deficit resulting in increased oxidisation of subcutaneous fat stores which coupled a decrease in extracellular fluid would result in the large body mass losses in male ultra-MTBers.

### Plasma urea, skeletal muscle damage, and protein catabolism

In male ultra-MTBers, post-race body mass was related to significant losses in post-race fat mass, decreases in extracellular fluid and increases in plasma urea (Table 4). Plasma urea increased in men by 108% (Table 3) and in women by 46.9%. In a 525-km cycling race, plasma urea rose significantly by 97% [37]. In another study investigating body composition and hydration status in one male ultra-endurance swimmer during a 24-hour swim, increases in plasma urea were associated with parameters of skeletal muscle mass damage [16]. We assume for the present male ultra-MTBers that the increase in plasma urea could be associated with skeletal muscle mass damage, because an increased plasma urea was related to changes in skeletal muscle mass in the present subjects. Nevertheless, due to the fact that absolute and percent changes in skeletal muscle mass were non-significantly, we assume that skeletal muscle mass damage was moderate in the present athletes. In contrast to cycling, Fellmann et al. demonstrated that a 24-hour running race caused more muscular lesions than a triathlon, where ultra-cycling was a part of the event [41]. After a Double Iron ultra-triathlon, plasma urea increased significantly [6] and indicated a state of protein catabolism of the organism in the athlete. Faster 24-hour ultra-MTBers in the present study showed increases in plasma urea, therefore a post-race increase in plasma urea may be attributed also to enhanced protein catabolism during ultra-endurance performance as was reported after an ultra-cycling race [39]. We speculate that an increase in plasma urea cannot be solely attributed to skeletal muscle damage and protein catabolism. Increased plasma urea in both sexes suggests an increased metabolic activity [64]. Plasma urea increases also in cases of an impaired renal function [39]. However, there was no association between the change in plasma urea and the change in urine specific gravity in both sexes in the present study.

### Race performance, fluid intake, and losses in body mass and fat mass

Despite the differences in the average cycling speed between women and men, men did not achieve a significantly higher number of kilometers during the 24 hours. Women may have on average shorter breaks during their race. Therefore, women were able to achieve a similar amount of kilometers as men. The better performance in the faster male and female ultra-MTBers could be also influenced by numerous reasons like the specific character of 24-hour races or good race tactics [18].

Another interesting finding was that in both male and female ultra-MTBers, faster finishers drank more than the slower ones, similarly as reported for 100-km ultra-marathoners [65]. Faster ultra-MTBers probably could have a higher sweating rate and lost more fluids, however total fluid intake was not related to changes in body mass, only to absolute ranking in the race in both sexes. Faster men and women showed also higher losses in body mass than slower ones, furthermore faster men lost more body fat than slower ones. Zouhal et al. [66] presented an inverse relationship between percent body weight change and finishing times in 643 forty-two-kilometer marathon runners. A decrease in body fat during an ultra-endurance triathlon was also associated with race intensity in ultra-triathletes [59]. Therefore, we assume that greater decreases in body mass seen here in male and female ultra-MTBers could be attributed to greater race intensity as well as decreases in fat mass in present male ultra-MTBers.

### Dehydration or overhydration in ultra-endurance performance?

Another important finding was the fact that foot volume remained stable in both sexes and no oedema of the lower limbs occurred in these ultra-MTBers. Moreover, the volume of the lower leg was neither related to fluid intake nor to changes in plasma [Na^+^]. This finding is in contrast with previous studies where an increased fluid intake was related to the formation of peripheral oedema [8,9]. Furthermore, fluid intake in the present study was not associated with changes in body mass, fat mass or plasma urea.

In case of a fluid overload we would expect an increase of solid mass and a decrease in plasma [Na^+^]. Fluid homeostasis in both sexes was relatively stable since haematocrit remained unchanged and plasma volume increased non-significantly. An increase in plasma volume in both groups may be due to [Na^+^] retention, as a consequence of an increased aldosterone activity [34]. Plasma [Na^+^] decreased only in men. Furthermore, the changes in plasma [Na^+^] were not related to the changes in plasma osmolality, or urine specific gravity. External factors such as compression socks might have an effect on running performance [67]. A recent study showed that male runners in a stepwise treadmill test improved running performance with the use of compression socks [67]. In the present study, 8 (21%) male 24-hour ultra-MTBers and 2 (17%) female 24-hour ultra-MTBers wore compression socks during the 24-hour race. Changes in total body water were non-significantly in both groups, and there were no differences in foot volume measured by plethysmography, so we did not assume that there was an accumulation of water with a subsequent extra-cellular oedema. On the contrary, during an intense performance in a hot environment, dehydration may occur [2], which may lead to a decrease in body mass [2,31], an increase in urine specific gravity [31], an increase in plasma and urine osmolality, and a decrease in total body water [43].

The present 24-hour ultra-MTBers appeared to have been relatively dehydrated since body mass decreased, however, as per definition of Noakes et al. [11] they were euhydrated. Urine specific gravity significantly increased in men where post-race urine specific gravity was 1.022 mg/L. Urine specific gravity > 1.020 mg/L is indicating significant dehydration according to Kavouras [43]. Urine specific gravity trended toward significance (1.020 mg/L) in women; they were minimally dehydrated according to Kavouras [43]. Urine specific gravity is considered as a reliable marker of hydration status [31,43], however, the change in urine specific gravity was very small and both pre- and post-race measurements were within the normal range limits [68] in both sexes. Moreover, the increase in urine specific gravity was not related to changes in body mass.

In both male and female ultra-MTBers, plasma osmolality did not reach post-race threshold value of 301 ± 5 mmol/kg, which is suggested [69] as a starting point for the estimation of the probability of dehydration. There was no association between percent changes in plasma osmolality and percent changes in plasma [Na^+^]; however, male finishers with an increased plasma osmolality had also increased plasma urea levels. The increase in plasma urea might lead to a change in plasma osmolality which might be a trigger for an increased activity of vasopressin [70]. Catabolic products of protein metabolism associated with a physical strain [3] could be also related to an increased urine osmolality, so it limits its potential utility for the assessment of dehydration. Similar limitations apply for urine specific gravity, and fluctuations in the volume of body fluid compartments will also affect plasma osmolality [3].

Prolonged exercise in the heat may cause increased losses of total body water by sweating and respiration [71]. However, total body water was stable in both sexes although extracellular fluid decreased significantly in men. The decrease in extracellular fluid in men was significantly and positively related to the change in body mass and significantly and negatively to the change in plasma urea. On the contrary, the change in extracellular fluid was not correlated to fluid intake or change in plasma volume. We assume that the present ultra-MTBers drank *ad libitum* and their average fluid intake was in line with the recommendation of the International Marathon Medical Directors Association (IMMDA) [72]. In the male ultra-MTBers, the decrease of extracellular fluid could be due to the race intensity accompanied by the reduction of the glycogen stores rather than due to dehydration. Ultra-MTBers in both sexes were not dehydrated, but they suffered a significant loss in solid masses.

### Limitations

The limitation was the relatively small number of female ultra-endurance ultra-MTBers. Probably a high energy deficit occurred during 24-hour races and we did not determine energy intake, in future studies it should be recorded.

### Practical applications for coaches and ultra-MTBers

Ultra-MTBers in both genders respond individualistically, although they had an equal access to fluid. These data support the finding that change in body mass during exercise may not reflect exact changes in hydration status, and higher losses of body mass did not impair race performance.

## Conclusions

To summarize, completing a 24-hour MTB race led to a significant decrease in total body mass and fat mass whereas skeletal muscle mass remained stable in both male and female competitors. The volume of the lower leg remained unchanged both in men and women. Body weight changes and increased plasma urea in both sexes under testing conditions do not reflect a change in body hydration, but rather represent a balance of both fluid and energy losses from both external and internal sources.

## Consent

Written informed consent was obtained from all testing subjects for the publication of this report and any accompanying images.

## Competing interests

The authors declare that they have no competing interests.

## Authors’ contributions

DCH, BK and TR developed the objectives of the study and intervention, DCH managed recruitment and data collection, TR supported a laboratory processing of samples, DCH and AZ participated in the practical measurement in all field studies, DCH and IT^4^ performed statistical analysis, DCH, BK and IT^4^ lead the drafting of the manuscript, interpreted the findings and critically reviewed the manuscript. MS helped with translation and the extensively correction of the whole text. All authors read and approved the final manuscript.
